# Integrating measurement and simulation for evaluation of patient‐specific whole‐body dosimetry in clinical BNCT

**DOI:** 10.1002/mp.70474

**Published:** 2026-05-10

**Authors:** Ryo Kakino, Naonori Hu, Akinori Sasaki, Mai Nojiri, Yuki Yoshino, Satoshi Takeno, Teruhito Aihara, Nishiki Matsubayashi, Takushi Takata, Hiroki Tanaka, Keiji Nihei, Koji Ono

**Affiliations:** ^1^ Kansai BNCT Medical Center Osaka Medical and Pharmaceutical University Takatsuki‐shi Osaka Japan; ^2^ Institute for Integrated Radiation and Nuclear Science Kyoto University Kyota Osaka Japan; ^3^ Department of Radiation Oncology Osaka Medical and Pharmaceutical University Takatsuki‐shi Osaka Japan; ^4^ Department of Radiology Kyoto Prefectural University of Medicine Kyoto Japan; ^5^ Department of Otorhinolaryngology ‐ Head and Neck Surgery Osaka Medical and Pharmaceutical University Takatsuki‐shi Osaka Japan; ^6^ BNCT Joint Clinical Institute Osaka Medical and Pharmaceutical University Takatsuki‐shi Osaka Japan

**Keywords:** boron neutron capture therapy, monte carlo simulation, out‐of‐field dose

## Abstract

**Background:**

Boron neutron capture therapy (BNCT) is a treatment modality that utilises high intensity neutron beam in combination with a boron drug to target cancer cells at the cellular level. Despite decades of research in this field, there are very few reports on out‐of‐field patient dose of BNCT.

**Purpose:**

To quantify patient whole‐body out‐of‐field dose during clinical BNCT for recurrent head‐and‐neck cancer using an integrated measurement–simulation workflow.

**Methods:**

The data of 271 patients that received BNCT for recurrent head and neck were analyzed for this study.First, the neutron and gamma ray doses were measured using activation foils and thermoluminescent dosimeters, respectively. The detectors were placed onto the surface of the patient at various locations and the reaction rate of the metal foils were measured. Second, PHITS Monte Carlo simulation was performed to evaluate the neutron energy spectrum at each location and the corresponding neutron equivalent dose was determined. The total dose was calculated by summing the neutron equivalent dose and the gamma ray dose. The results were compared with other radiotherapy modalities.

**Results:**

The mean ± standard deviation of equivalent dose at neck, chest, abdomen, waist, knee, and ankle were calculated to be 1.93 ± 1.12, 0.71 ± 0.42, 0.23 ± 0.12, 0.11 ± 0.06, 0.05 ± 0.03, 0.02 ± 0.01 Gy‐eq, respectively. Excluding the neck region, the gamma ray dose was dominant at all measurement points. From the abdomen below, dose from the primary gamma ray were dominant, indicating a potential for reducing the dose to these regions by installing additional lead shielding in the treatment room walls. In comparison to other radiotherapy modalities, out‐of‐field dose of BNCT was found to be similar.

**Conclusion:**

The whole‐body dose for patients that received BNCT for recurrent head and neck cancer were estimated by scaling PHITS derived neutron dose using multifoil reaction rates and adding measured gamma dose. The results showed the out‐of‐field dose was comparable to other radiotherapy modalities and further added confidence that BNCT is a safe treatment modality.

## 1 INTRODUCTION

Boron neutron capture therapy (BNCT) is a binary radiotherapy technique based on nuclear reactions that occur when ^10^B atoms capture thermal neutrons, producing particles with high linear energy transfer (LET) that deposit their energy over several micrometers—approximately the diameter of a typical human cell. This treatment has been studied mainly for brain and head and neck regions.^1^, ^2^, ^3^ BNCT has traditionally been performed using nuclear reactors, but recent years have seen significant development in accelerator‐based neutron sources (ABNS) worldwide.

The world's first commercially available ABNS for clinical BNCT, developed by Sumitomo Heavy Industries (NeuCure® BNCT System), along with its dose calculation system (NeuCure® dose engine), received manufacturing and marketing approval as a new medical device from the Ministry of Health, Labor and Welfare in March 2020. BNCT for unresectable, locally advanced, and recurrent carcinoma of the head and neck cancer was approved by the Japanese government for reimbursement under the national health insurance in June 2020. More than 300 cases of BNCT have been performed in our institution as of July 2024. The preliminary outcome of 69 patients (72 cases) during the first two years have been reported by Takeno et al.^4^

Out‐of‐field dose is an important concern for patient safety, regardless of the treatment modality. This dose is categorized as a “nontarget” dose and is problematic in relation to secondary cancers (in long‐term prognosis),^5^ cardiac toxicity,^6^, ^7^ failure of implantable pacemakers and other electronic devices,^8^, ^9^ pregnant patients,^10^, ^11^ cataracts,^12^, ^13^ and skin damage,^14^, ^15^ although the dose levels can vary.^16^ In photon therapy, the main components of the out‐of‐field dose are: (a) collimator scatter, (b) patient scatter, and (c) head leakage. Head leakage becomes more dominant the further away it is from the irradiation field. In BNCT, the patient's exposure to an out‐of‐field dose leaked from the collimator wall might be high because the patient must be positioned as close to the collimator wall as possible. Unfortunately, AAPM TG‐158 does not address BNCT. To ensure the safety of BNCT, it is important to evaluate the whole‐body dose in patients who have undergone BNCT and to devise ways to reduce it.

At our institution, whole‐body exposures have been measured for all patients who underwent BNCT, using the multifoil activation method for neutron measurement and thermoluminescent dosimeters (TLDs) for gamma‐ray measurement (details are provided in the Methods section). Gamma‐ray doses can be evaluated directly, whereas it is difficult to directly convert the reaction rate obtained from neutron measurements into dose, as the reaction rate represents an integral over the neutron energy spectrum. Meanwhile, our group has developed the foundation for a BNCT dose calculation system. First, we commissioned a treatment planning system (TPS) used for clinical BNCT (the NeuCure^®^ dose engine with RayStation) and developed an independent Monte Carlo simulation model based on the Particle and Heavy Ion Transport code system (PHITS).^17^ The PHITS model can be used for fundamental research to calculate BNCT doses under conditions that are not feasible with the commercial TPS, such as calculating neutron reaction rates for activation foils,^18^ developing new collimator designs,^19^ and performing dose calculations based on CT values.^20^ Furthermore, the PHITS model can now be applied to the whole‐body region by extending the source and tally regions.^21^ The measured reaction rates, combined with the PHITS model, may enable the estimation of whole‐body doses for each patient. Therefore, the purpose of this study was to evaluate the whole‐body dose during BNCT by integrating measurement and simulation.

## 2 METHODS

**FIGURE 1:**
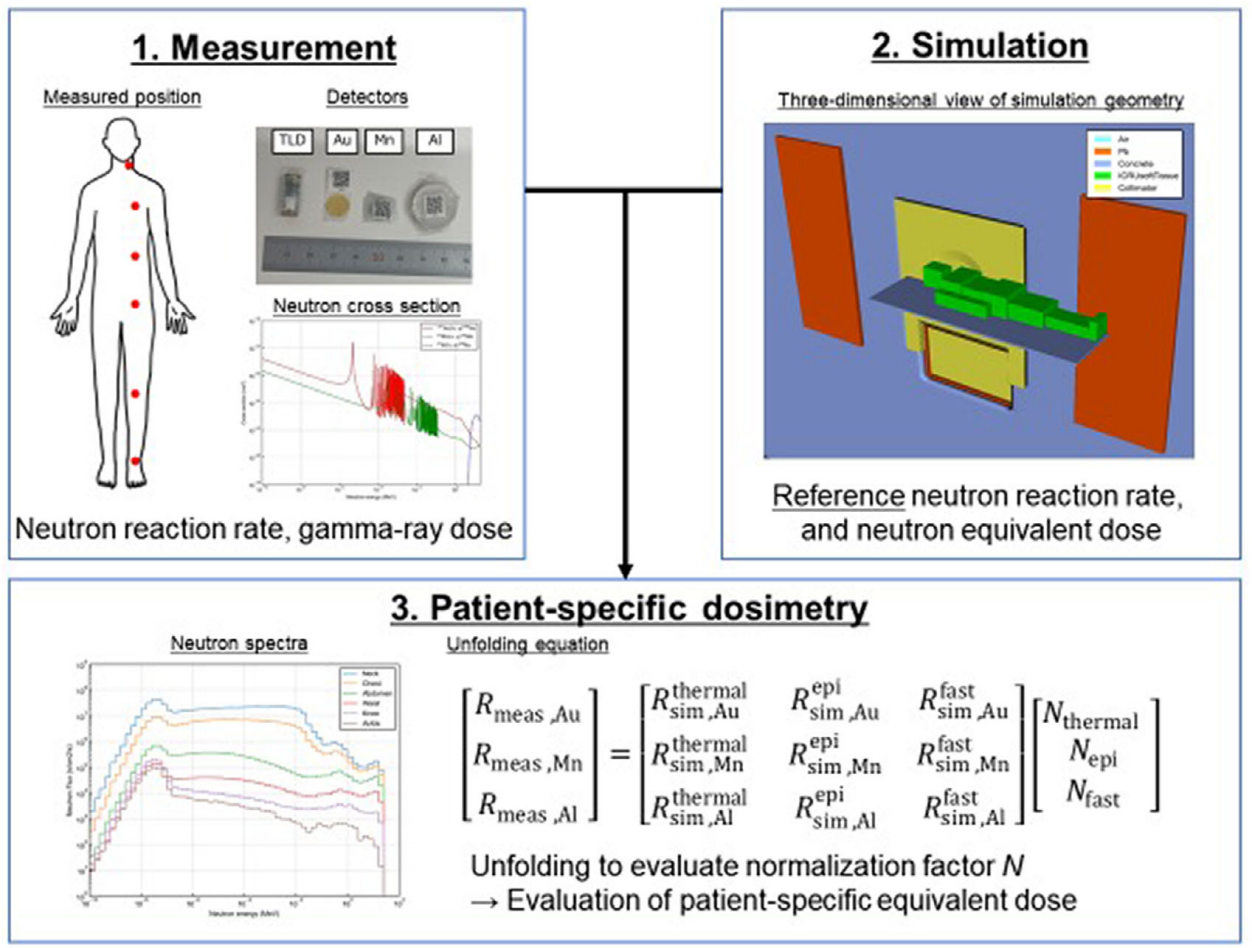
The overall methodology of this study. By combining the measurement and simulation, patient‐specific whole‐body dose can be evaluated.

The overall methodology used in this study is illustrated in Figure 1. The neutron reaction rate and gamma‐ray dose were measured for each patient. Since the neutron reaction rate cannot be directly converted into dose, a simulation was conducted to obtain a “reference” neutron reaction rate and its equivalent dose. By combining the measurement and simulation, patient‐specific whole‐body dose can be evaluated. The details of each section are described below.

### 2.1 Patients

This retrospective study was approved by the Institutional Review Board at our institution (approval number: No. 2021–172). We considered all the head‐and‐neck cancer patients who underwent BNCT at our institution from June 2020 to March 2024. Patients for whom measurement results were unavailable at any measurement point were excluded. As a result, 271 out of 290 patients were eligible for inclusion in this study.

### 2.2 Measurements

**FIGURE 2:**
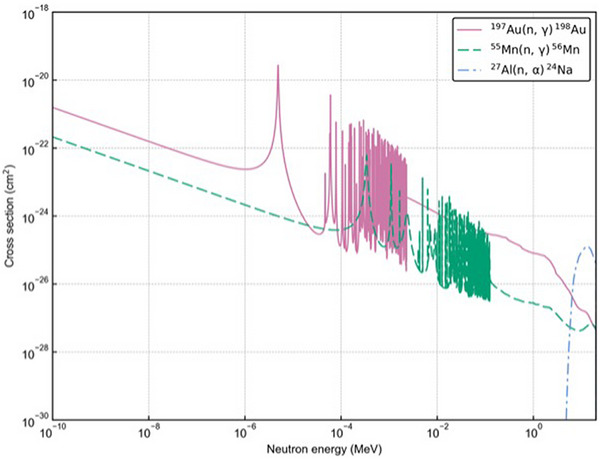
Neutron cross sections of multifoils used in the experiment.

The multifoil activation method^22^, ^23^ was applied to neutron measurements for each patient. Gold (10 mm diameter, 0.05 mm thickness), manganin (10 mm diameter, 0.2 mm thickness, containing approximately 13 wt% of manganese), and aluminium (20 mm diameter, 2.5 mm thickness) foils (referred as Au, Mn, and Al, respectively, The Nilaco Corporation) were utilized for neutron detection. These foils are sensitive to the different neutron energy regions: thermal and epi‐thermal (Au), thermal (Mn), and fast neutron (Al), respectively. Their neutron cross sections are summarized in Figure 2. Photon doses were measured using TLDs, specially ordered BeO powder enclosed in quartz glass capsules (Panasonic UD‐170LS).^24^ Before the irradiation, the four dosimeters were placed at six locations on the side of each patient closer to the beam port: neck, chest, abdomen, waist, knee, and ankle. After irradiation, the gamma‐ray peaks emitted from the neutron detectors (Au: 412 keV, Mn: 847 keV, Al: 1369 keV) were measured using a high‐purity germanium (HPGe) detector (ORTEC ICS‐P4). The measured counts were then converted to the reaction rate per unit charge per atom (n/C/atom) for each neutron detector. Details of the conversion process can be found elsewhere.^21^, ^25^

### 2.3 Simulation

**FIGURE 3:**
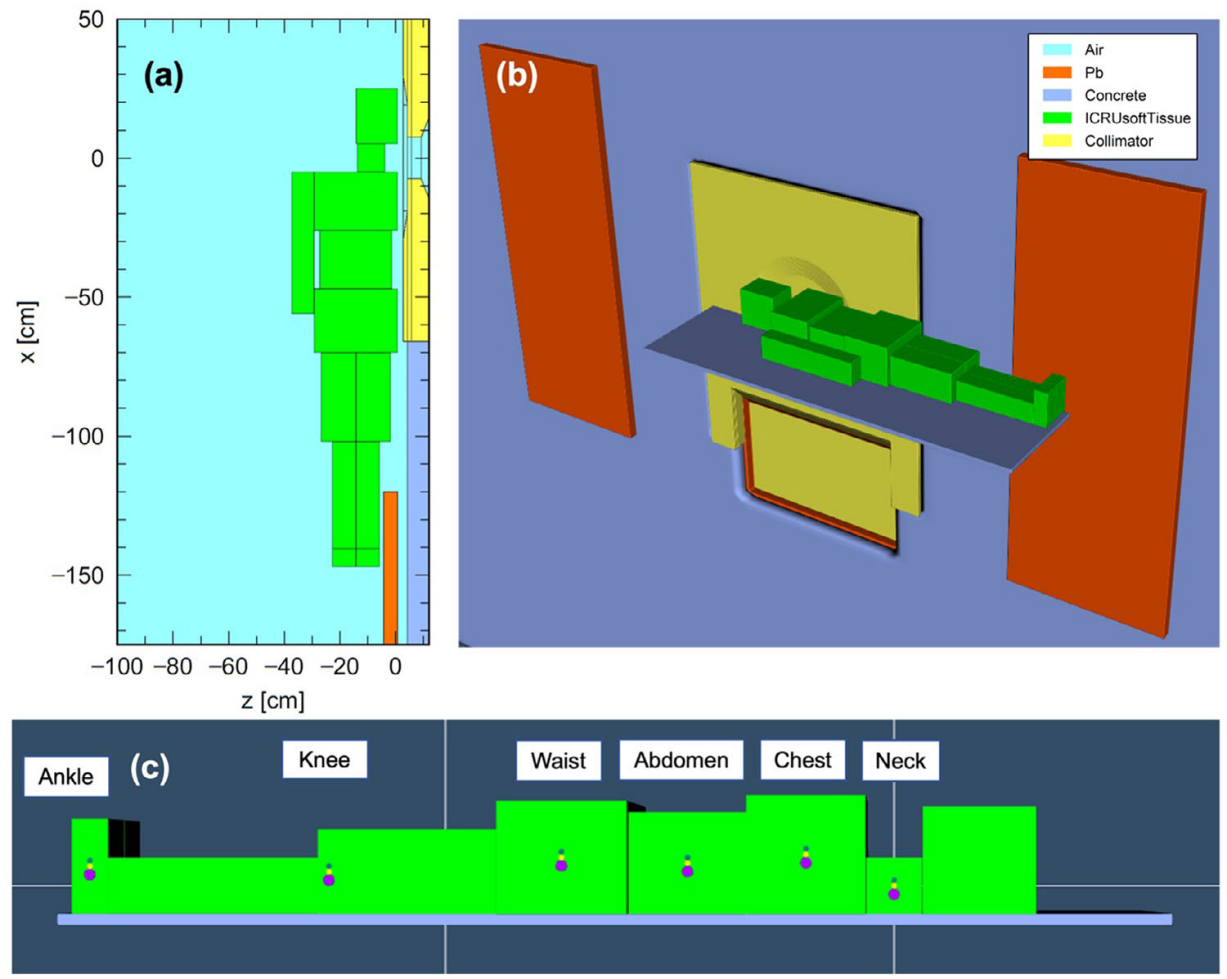
Arrangement of the human‐body phantom in the PHITS simulation: (a) coronal view, (b) 3D view, and (c) beam‐eye view with multifoils.

PHITS Monte Carlo simulation was utilized to evaluate the neutron equivalent dose based on the measured reaction rates. The PHITS model for the entire BNCT irradiation room had already been validated in our previous study.^21^ In this model, a human‐body phantom was created by combining several rectangular block phantoms, as shown in Figure 3. The phantom material was assigned as ICRU soft tissue (elemental composition by weight, *H*: 10.1%, C: 11.1%, N: 2.6%, O: 76.2%) with a ^10^B concentration of 25 ppm. The region within 0.07 mm from the surface of the phantom was defined as the skin. The dosimeters described in the previous section were modeled as shown in Figure 3 (c). The equivalent dose at skin due to neutrons (denoted as *D*_sim_) at each location and the reaction rates (*R*_sim_) of each activation foil and the neutron energy spectrum, were simulated with the [*T*‐track] function in PHITS. The simulated dose, *D*_sim_, was calculated using the following equation:

(1) $$D_{\mathrm{sim}}=CBE\times D_{B}+RBE_{N}\times D_{N}+RBE_{H}\times D_{H}$$

where CBE is compound biological effectiveness (set to 2.5 for skin), RBE_N_ and RBE_H_ are the relative biological effectiveness values for nitrogen and hydrogen (2.9 and 2.4, respectively, for NeuCure^®^), *D_B_*, *D_N_*, *D_H_* are the physical doses from boron, nitrogen, and hydrogen, respectively. *C*_B_ is the ^10^B concentration measured in the blood samples from each patient who underwent BNCT.

The neutron energy range was set to:

- Thermal neutron range: 1 × 10–20 MeV < En ≤ 5.3 × 10–7 MeV
- Epi‐thermal neutron range: 5.3 × 10–7 MeV < En ≤ 1 × 10–2 MeV
- Fast neutron range: 1 × 10–2 MeV < En ≤ 30 MeV

### 2.4 Methodology for evaluating neutron equivalent dose

The ratio of the measured and simulated reaction rates was defined as the normalization factor *N* ( = *R*_meas_ / *R*_sim_) for the neutron equivalent dose in each neutron energy region. The relationship between *R*_meas_ and *R*_sim_ is expressed by the following equation:

(2) $$\left[\begin{array}{c} R_{\mathrm{meas},\mathrm{Au}}\\ R_{\mathrm{meas},\mathrm{Mn}}\\ R_{\mathrm{meas},\mathrm{Al}} \end{array}\right]=\left[\begin{array}{ccc} R_{\mathrm{sim},\mathrm{Au}}^{\mathrm{thermal}} & R_{\mathrm{sim},\mathrm{Au}}^{\mathrm{epi}} & R_{\mathrm{sim},\mathrm{Au}}^{\mathrm{fast}}\\ R_{\mathrm{sim},\mathrm{Mn}}^{\mathrm{thermal}} & R_{\mathrm{sim},\mathrm{Mn}}^{\mathrm{epi}} & R_{\mathrm{sim},\mathrm{Mn}}^{\mathrm{fast}}\\ R_{\mathrm{sim},\mathrm{Al}}^{\mathrm{thermal}} & R_{\mathrm{sim},\mathrm{Al}}^{\mathrm{epi}} & R_{\mathrm{sim},\mathrm{Al}}^{\mathrm{fast}} \end{array}\right]\;\left[\begin{array}{c} N_{\mathrm{thermal}}\\ N_{\mathrm{epi}}\\ N_{\mathrm{fast}} \end{array}\right]$$

The matrix of *R*_sim_ on the right side was calculated using the PHITS simulation described above. From this equation, the normalization factors *N* can be estimated through a simple unfolding process using inverse matrix of *R*_sim_. The neutron equivalent dose can then be estimated using the following equation:

(3) $$D_{n}=\sum_{j}N_{j}{\left(D_{\mathrm{sim}}\right)}_{j}\;$$

where *j* denotes the neutron energy region. The total equivalent dose was obtained by adding the gamma‐ray dose to the neutron equivalent dose. The evaluated neutron equivalent dose was then compared with those from other modalities, as referenced in AAPM TG‐158. According to TG‐158, the out‐of‐field dose is evaluated by dividing the dose equivalent (Sv) by the prescribed target dose (Gy). In BNCT, the prescribed dose is typically based on the clinically tolerable limit of the organs at risk (OARs, such as mucous membrane, skin, brain, or optic nerve), but it varies from patient to patient due to the location of the OAR with respect to the tumor. Although a direct comparison with other modalities is difficult, the neutron dose equivalent was divided by the dose received by at least 95% of the tumor volume (D_95%_), as this metric is commonly used for dose prescription in modern radiotherapy.^26^ Here, the tumor dose was calculated by assuming a tumor to blood ratio of 2.5 and a CBE of 3.8.

### 2.5 Simulation for searching the origin of gamma‐rays

It has been reported that the gamma‐ray dose contributes more to the whole‐body dose in BNCT than the neutron dose.^27^ This suggests that gamma‐ray shielding materials on the Beam‐Shaping Assembly (BSA) side may be effective in reducing whole‐body dose. However, the origin of gamma‐rays is not limited to the BSA side (i.e., primary gamma‐rays); secondary gamma rays are also produced within the patient's body through the ^1^ *H*(*n*, γ)^2^H reaction. If the contribution of the secondary gamma‐ray dose exceeds that of the primary component, shielding on the BSA side may be ineffective. To investigate the origin of the gamma‐ray dose, the sources of neutrons and photons were separately tallied within the human‐body phantom using the [*T*‐track] function.

### 2.6 Comparison of dose equivalent between BNCT and other modalities

The out‐of‐field whole‐body doses in BNCT were compared with those in other treatment modalities. AAPM TG‐158 provides a comprehensive summary of out‐of‐field doses normalized to the prescription dose (mSv/Gy) for photon, proton, carbon ion, and other radiotherapy modalities. Although TG‐158 recommends the use of biologically weighted dose when RBE data are available, many studies have adopted dose equivalent or ambient dose equivalent H*(10) for inter‐modality comparisons. Therefore, to enable comparison with other modalities from a radiological protection perspective, the H*(10) for each patient was additionally calculated separately. Neutron H*(10) was evaluated using PHITS by applying fluence‐to‐ambient dose equivalent conversion coefficients to the calculated neutron fluences provided in ICRP Publication 74. The total H*(10) was obtained by adding the gamma‐ray dose measured by TLD to the neutron H*(10), assuming that 1 Gy = 1 Sv for gamma‐rays. Finally, the total H*(10) was normalized to the delivered equieffective dose in 2 Gy fractions (EQD2) of D_95%_ at the tumors for each patient (Sv/Gy‐eq) using the linear‐quadratic (LQ) model:

(4) $$EQD2=nd\times \frac{\left(d+\alpha /\beta \right)}{\left(2+\alpha /\beta \right)}$$

where n is the number of fractions, d is the dose per fraction, and α/β = 10 Gy for tumor. D_95%_ was selected as the normalization dose because it is a standard metric of target coverage in radiation therapy. This normalization enables comparison across modalities despite differences in fractionation schedules. Additionally, the biologically weighted dose evaluated in the previous section was also converted to EQD2 (a/b = 2 Gy) and then normalized to the delivered EQD2 of D_95%_ at the tumors for each patient.

## 3 RESULTS

**FIGURE 4:**
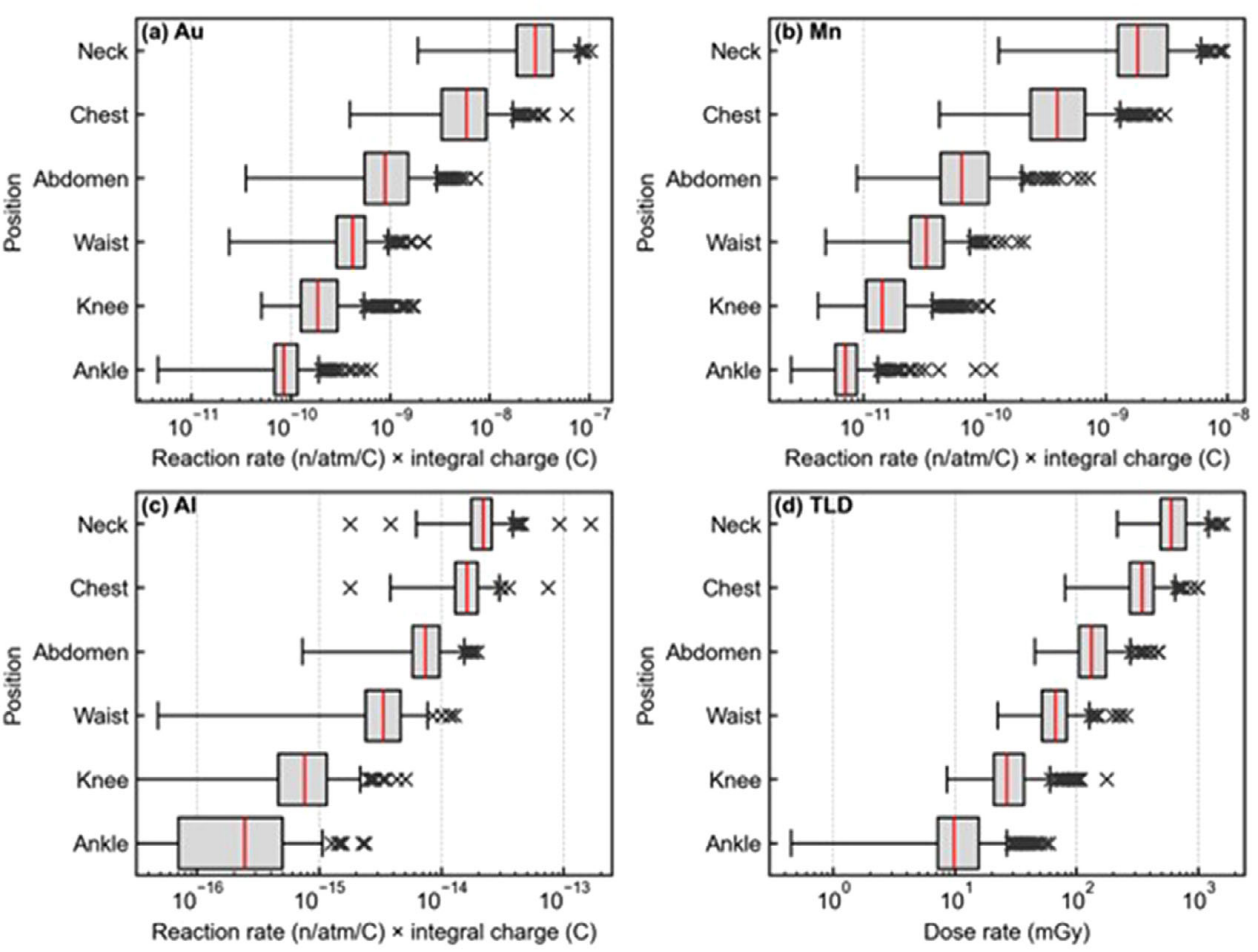
The measured reaction rate at each body location for (a) Au, (b) Mn, and (c) Al. The gamma ray absorbed dose measured with the TLD is shown in (d).

**FIGURE 5:**
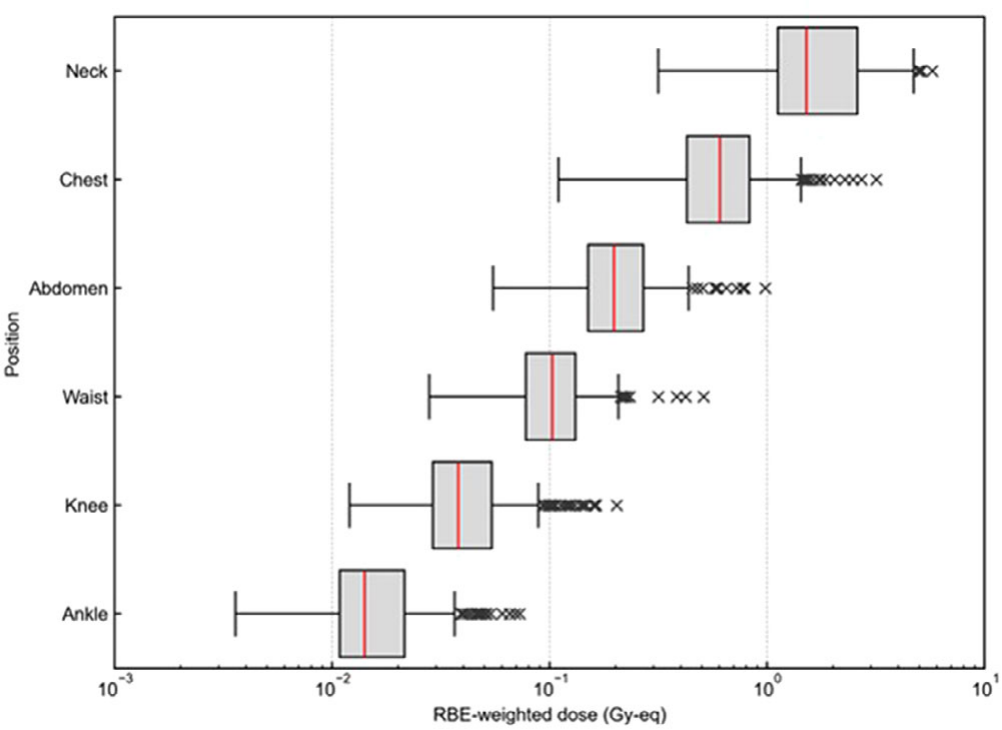
Whole‐body doses at each body locations.

**FIGURE 6:**
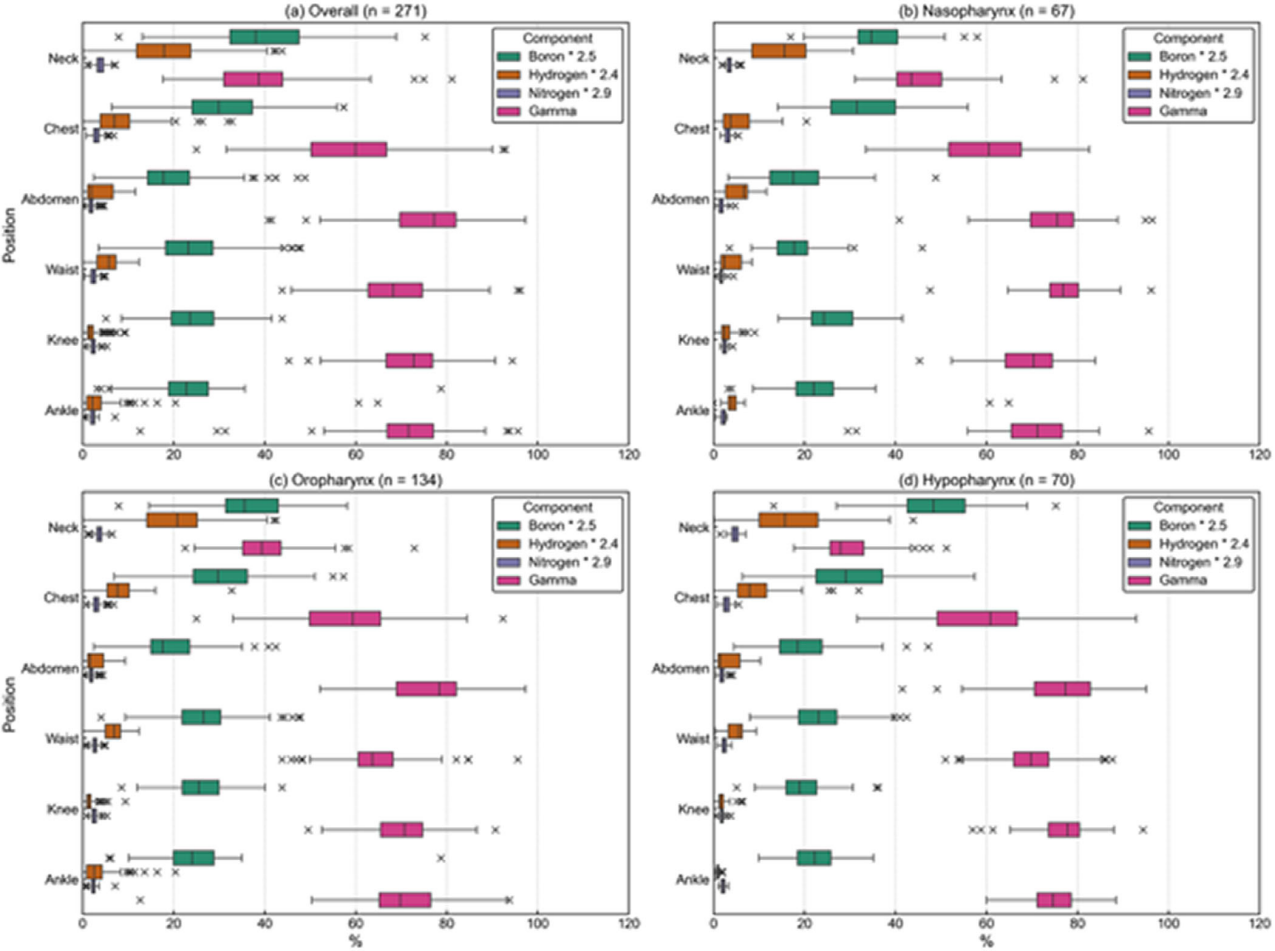
Ratios of dose components at each body location categorized into different treatment sites. (a) Overall patients, (b) Nasopharyngeal cancer/Nasal cavity/Paranasal sinus region, (c) Oropharyngeal/Lip and oral cavity/Salivary gland region, (d) Hypopharyngeal/Laryngeal region.

Figure 4 shows the measured reaction rate of each metal foil at each body location and the gamma ray dose. All measurement points showed a similar trend where the reaction rate and gamma ray dose was the highest near the field. Figure 5 shows the whole‐body equivalent dose at each measurement location. The mean ± standard deviation of equivalent dose at the neck, chest, abdomen, waist, knee, and ankle were 1.93 ± 1.12, 0.71 ± 0.42, 0.23 ± 0.12, 0.11 ± 0.06, 0.05 ± 0.03, 0.02 ± 0.01 Gy‐eq, respectively. When the CSD was increased by 3 cm, the corresponding values were 1.98 ± 1.17, 0.68 ± 0.43, 0.22 ± 0.12, 0.11 ± 0.05, 0.04 ± 0.03, 0.02 ± 0.01 Gy‐eq, respectively. The absolute differences were within 0.03 Gy‐eq at all locations, indicating that the dose evaluation is robust against variations in patient locations. Figure 6 summarizes the ratios of dose components at each location, categorized into three major treatment areas (nasopharyngeal, oropharyngeal, and hypopharyngeal). Among the four dose components, the gamma‐ray dose was dominant at all measurement points except for the neck.

**FIGURE 7:**
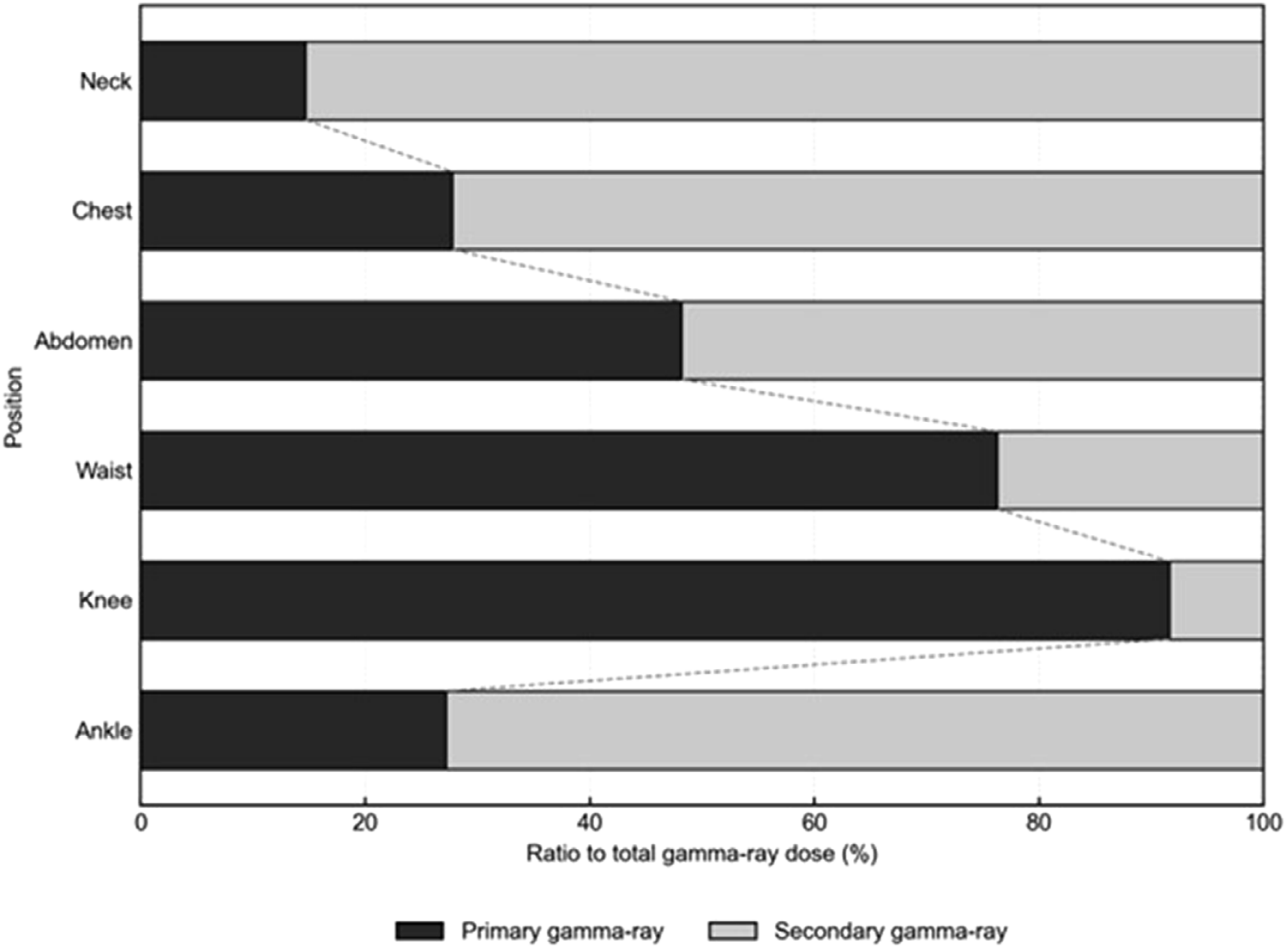
The ratio of primary and secondary gamma‐ray dose to all gamma‐ray dose for each calculated point.

Figure 7 shows the ratio of primary and secondary gamma‐ray doses to total gamma‐ray dose at each measurement point. The ratio of primary gamma‐rays increased with distance from the center of the irradiation field, while the ratio of secondary gamma‐rays was highest at the ankle position.

**FIGURE 8:**
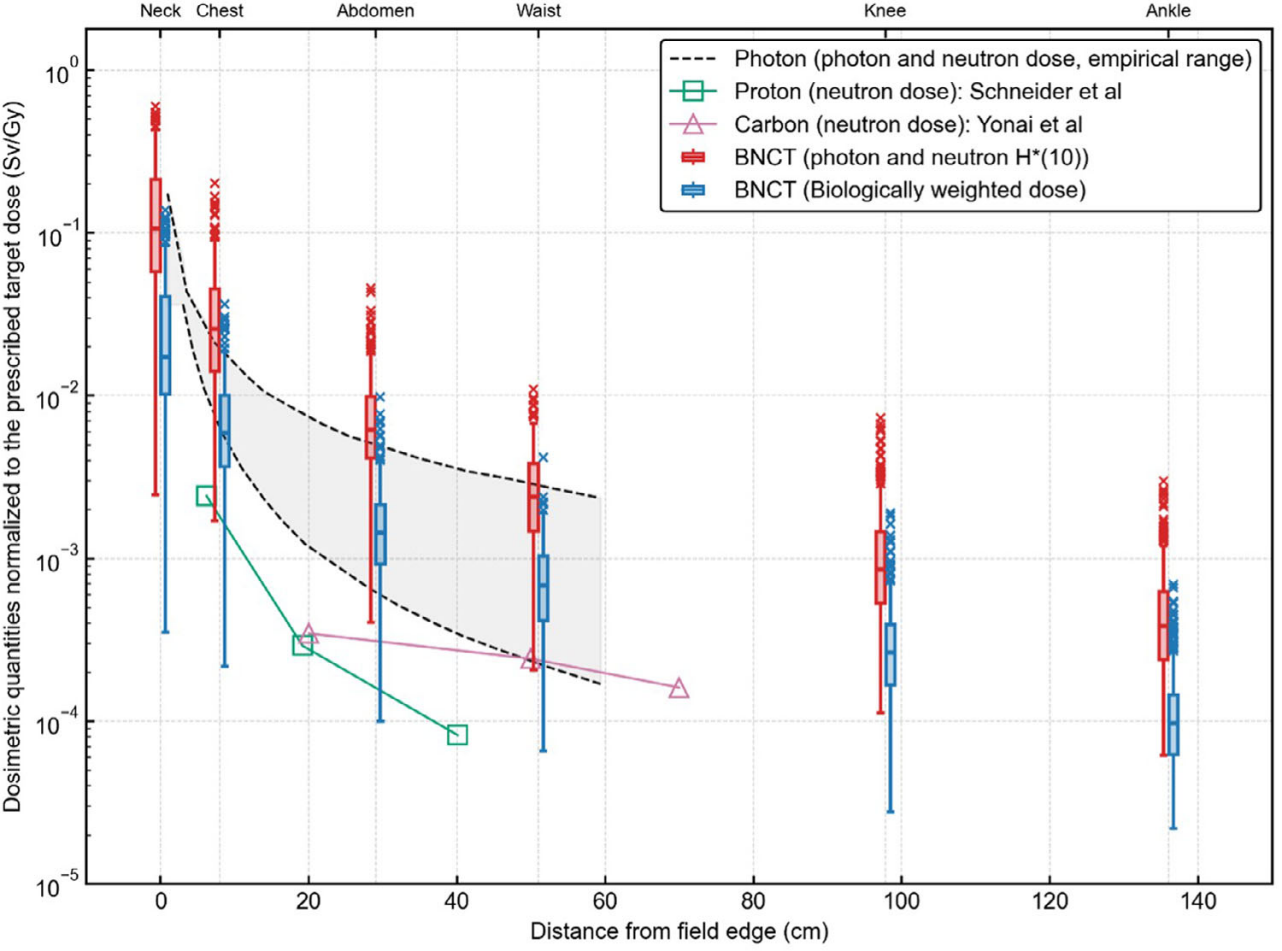
Comparison of dose equivalent per prescribed dose as a function of distance from the field edge between BNCT evaluated in this study and other modalities (photon, proton, and carbon) described in AAPM TG‐158.

The evaluated doses were compared with the summary of out‐of‐field dose reported in the AAPM TG‐158, as shown in Figure 8. Evaluated absorbed dose, biologically weighted dose and H*(10) for each location are summarized in Table 1.

**TABLE 1:** Evaluated absorbed dose, biologically weighted dose and H*(10) for each location.

Region	Gamma‐ray absorbed dose (Gy)	Neutron absorbed dose (Gy)	Total absorbed dose (Gy)	Biologically weighted dose (Gy‐eq)	H*(10) (Sv)
Neck	0.65 ± 0.25	0.52 ± 0.37	1.18 ± 0.59	1.95 ± 1.13	6.77 ± 4.90
Chest	0.36 ± 0.13	0.17 ± 0.12	0.53 ± 0.24	0.79 ± 0.42	1.88 ± 1.25
Abdomen	0.15 ± 0.07	0.03 ± 0.02	0.18 ± 0.09	0.23 ± 0.12	0.50 ± 0.25
Waist	0.07 ± 0.03	0.02 ± 0.01	0.09 ± 0.04	0.11 ± 0.06	0.18 ± 0.09
Knee	0.03 ± 0.02	0.01 ± 0.01	0.04 ± 0.02	0.05 ± 0.03	0.07 ± 0.05
Ankle	0.01 ± 0.01	0.00 ± 0.00	0.02 ± 0.01	0.02 ± 0.01	0.03 ± 0.03

## 4 DISCUSSION

The empirical range of photon therapy doses shown in Figure 8 includes various treatment techniques (segmental, dynamic MLC, and VMAT), energies (6, 10, and 18 MV), treatment sites (brain, head‐and‐neck, and prostate), and different depths within the phantom. These doses were measured using TLDs, radiochromic films, and ionization chambers for photons and gold foils for neutrons.^28^, ^29^, ^30^, ^31^, ^32^ In spot‐scanning proton therapy, Schneider et al. measured the dose equivalent of secondary neutrons using Bonner sphere and CR39 etch detectors, with calculations performed by the FLUKA code.^33^ In heavy ion therapy, Yonai et al. measured the dose equivalent of secondary neutrons using a tissue‐equivalent proportional counter (TEPC) inside a water phantom.^34^ In this study, the whole‐body dose was evaluated for 271 head‐and‐neck cancer patients who underwent BNCT at our institution. To the best of our knowledge, no previous study has evaluated whole‐body dose for such a large clinical BNCT patient dataset, although several prior studies have simulated whole‐body dose using computational phantoms or whole‐body CT images.^35^, ^36^, ^37^ The results of this study indicate that the whole‐body dose in BNCT is comparable to that of photon, proton, and carbon ion therapies, supporting the safety profile of BNCT. In this study, both the biologically weighted dose and the H*(10) for BNCT were evaluated by integrating measurement and simulation. These represent fundamentally different dosimetric concepts. The biologically weighted dose, the preferred quantity in AAPM TG‐158 when RBE data are available for non‐target dose assessment, reflects the RBE of each radiation component and is directly relevant to clinical risk estimation. In contrast, H*(10) is an operational quantity defined for radiation protection purposes, representing the dose equivalent at a depth of 10 mm in the ICRU sphere under free‐field irradiation conditions. Although, the H*(10) values in BNCT were slightly higher than the empirical range of those for photon therapy, direct comparison should be interpreted with caution. First, H*(10) is inherently a free‐field quantity, whereas the neutron fluence spectra in this study were evaluated at the patient body surface rather than at organ depths within the patient. As noted in TG‐158, surface assessments substantially overestimate organ doses due to the steep depth‐dose dependence of neutrons. Second, the photon therapy data compiled in TG‐158 were predominantly measured or calculated at depths within phantoms corresponding to organ positions, meaning that the comparison involves inherently different evaluation depths. For these reasons, the H*(10) values reported here should be regarded as conservative estimates.

The contribution of boron, nitrogen, hydrogen, and gamma‐ray doses to the patient‐specific whole‐body equivalent dose was dominated by the gamma‐ray dose at all measurement points except the neck, a finding consistent with Tsukamoto et al^27^ Furthermore, simulation results showed that the ratio of primary to secondary gamma‐ray dose components at each measurement point increased with lateral distance from the irradiation center, with the primary component reaching 91.6% at the knee. Although the primary gamma‐ray dose ratio at the ankle was relatively low (27.2%), this is likely due to shielding by the lead shutter near the ankle, as shown in Figure 3. As demonstrated in the Appendix, the neutron dose equivalent decreases with increasing distance from the irradiation field, whereas the gamma‐ray dose equivalent remains relatively constant. Therefore, by applying high‐Z materials such as lead on the BSA side, it is possible to effectively reduce the whole‐body dose, which is expected to help minimize adverse events in patients undergoing BNCT.

The evaluated equivalent dose varied by less than approximately 25% due to shifting the human‐body phantom within ± 75 mm. The neck and chest positions are regions where the lateral neutron intensity changes significantly. Therefore, the reference neutron reaction rate at these points may have been substantially affected by the phantom shift, which in turn influenced the evaluated equivalent dose. The waist position (located about –65 cm laterally from the irradiation center) corresponds to the boundary on the BSA side between the LiF PE and the concrete; the intensity variation at this boundary contributed to fluctuations in the equivalent dose. At other measurement points, lateral intensity variations were relatively small, resulting in smaller fluctuations in the equivalent dose. Since patient positioning varies depending on the target site and the patient's performance status, it would be ideal to deform the human‐body phantom shape for each individual patient. However, this is not practical for routine clinical work. Therefore, it should be noted that our proposed method may introduce potential errors due to the fixed phantom position.

There is a limitation in whole‐body dosimetry using activation foils. At our institution, gamma‐rays emitted from aluminum foils are routinely measured using an HPGe detector for 30 min per sample. The gamma‐ray peak counts at the neck position typically reach several thousand per 30 min, but only a few counts per 30 min at the ankle position. This low count at the ankle is due to the relatively small neutron cross section of the ^27^Al(*n*, *α*)^24^Na reaction with the threshold of 3.4 MeV, resulting in higher statistical uncertainty in fast neutron measurements. Additionally, the long measurement time presents a bottleneck for routine clinical operations. Although indium foils are also standard for fast neutron measurement (^115^In(*n*, *n*’)^115m^In), their exceptionally large cross section of ^115^In(*n, γ*)^116m^In generates high induced radioactivity which causes dose enhancement to the patients. As alternative methods for fast‐neutron detection, nickel could be utilized via the ^5^ ^8^Ni(*n*,*p*)^5^ ^8^Co reaction, but the long half‐life of ^5^ ^8^Co (70.9 days) makes it difficult for routine clinical use. Moderation‐based methods would perturb the counts of Au and Mn, so they would not be applicable to the current workflow. Outside of activation methods, bubble detectors and CR‐39 solid‐state nuclear track detectors are useful for fast‐neutron detection, but both require cumbersome processing after BNCT, making them difficult for routine clinical use. On the other hand, the contribution of fast‐neutron‐induced hydrogen dose to the total biologically weighted dose was approximately 10% or less. Furthermore, the biologically weighted dose at the knee and ankle was evaluated to be approximately below 0.1 Gy‐eq. Most OARs are not located at these sites. Therefore, patient‐specific measurement of fast neutrons at distal locations would not be as important from the perspectives of radiation oncology and radiation protection. A more practical approach for far locations (e.g., more than 70 cm from the field edge) would be to acquire reference dose data across all neutron energy regions on the collimator wall.

## 5 CONCLUSION

The whole‐body dose for patients who received BNCT for recurrent head and neck cancer was evaluated by combining experimental measurements with Monte Carlo simulation results. The findings showed that the out‐of‐field dose was comparable to that of other radiotherapy modalities, providing further confidence that BNCT is a safe treatment option.

## FUNDING INFORMATION

This work was supported in part by the JSPS KAKENHI grant numbers JP21K20521 and JP24K18810.

## CONFLICT OF INTEREST STATEMENT

The authors declare no conflicts of interest.

## Figures and Tables

**FIGURE A1:**
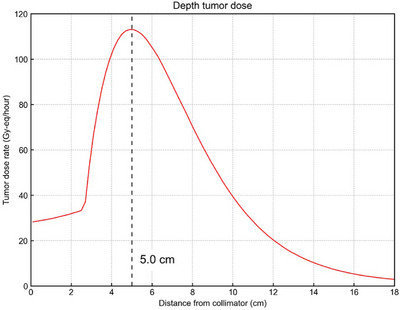
Depth tumor dose from the neutron beam exit at the central axis of the phantom for the reference collimator (12 cm in diameter).

**FIGURE A2:**
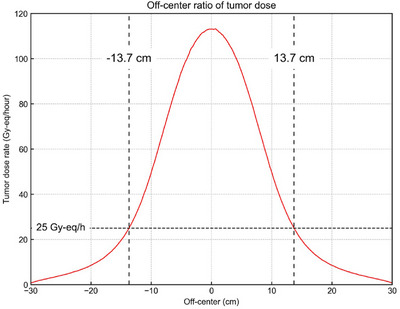
Off‐center ratio of tumor dose at the depth of the maximum tumor dose in the phantom for the reference collimator (12 cm in diameter). The field edge was defined as the off‐center distance at where the tumor dose rate was equal to 25 Gy‐eq/h.

**FIGURE A3:**
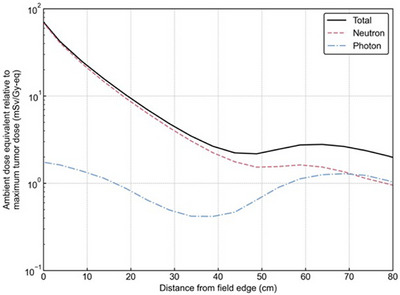
Neutron and gamma‐ray ambient dose equivalent from the field edge for the reference collimator (12 cm in diameter).

## APPENDIX A Numerical stability of the rsim matrix for the unfolding problem.

### A.1

For evaluating the numerical stability of the unfolding procedure, the condition numbers of ***R*_sim_** [*κ*_2_(***R*_sim_**)] for each measuring position were calculated by the following equation:

(A1)
$$\kappa_{2}\;\left(R_{sim}\right)=\frac{\sigma_{max}}{\sigma_{min}}$$

where $\sigma_{max}$ and $\sigma_{min}$ are the maximum and minimum singular values of *R_sim_*, respectively, which are evaluated by the singular value decomposition. Table A1 shows the condition numbers for each measuring location. To ensure the stability and non‐negativity of the evaluated dose, the unfolding procedure was performed by Regularized Non‐Negative Least Squares, as follows:

(A2)
$$\;\min_{n\geq 0}∥R_{\mathrm{sim}}n-r_{\mathrm{meas}}{∥}^{2}+\alpha^{2}∥n-n_{\mathrm{pri}}{∥}^{2},$$

where *α* is the regularization parameter (set to 10^−7^), and ***n*_pri_** is the all‐ones vector multiplied by the irradiation time in seconds for each patient.

**TABLE A1:** Condition numbers of *R*_sim_.

Body region	Condition number
Neck	1.59 × 10^6^
Chest	2.10 × 10^5^
Abdomen	2.32 × 10^4^
Waist	3.87 × 10^3^
Knee	2.89 × 10^4^
Ankle	2.15 × 10^5^

## APPENDIX B Out‐of‐field dose.

### B.1

IAEA TECDOC report “Advances in Boron Neutron Capture Therapy” recommends evaluating the ambient dose equivalent (*H**(10)) of neutrons and photons at distances between 15 and 200 cm from the field edge^38^; the parameters and conditions for the evaluation are summarized in the report. Following this, a cubic phantom of 60 cm per side set to ICRU soft tissue (elemental composition [wt%], *H*: 0.101, C: 0.111, N: 0.026, O: 0.762) was modeled using PHITS. The position of the phantom was explored on the central axis, where the distance between the beam exit and maximum depth of tumor dose in the phantom was 5 cm. The maximum tumor dose (Gy‐eq) under the condition on the central axis was used to normalize the *H**(10) (Sv/Gy‐eq). Then, the *H**(10) in air was simulated in off‐central direction at 5 cm from the beam exit for the reference collimator (12 cm diameter).

When the distance from the beam exit to the phantom surface was set to 2.7 cm, the maximum tumor dose depth was 5 cm, as shown in Figure A1. The field edge was evaluated to be 13.7 cm (off‐central distance where the tumor dose rate was calculated to be 25 Gy‐eq/h), as shown in Figure A2. Figure A3 shows the neutron and photon ambient dose equivalent from the field edge. The neutron dose equivalent decreased almost monotonically as the distance increased; the gamma‐ray dose equivalent decreased up to 40 cm, then increased up to 70 cm, reaching 72% of the dose at 0 cm.

## APPENDIX C Uncertainty sources and propagation in dose evaluation.

### C.1

The dose reported above as mean ± standard deviation represents the observed variance among patients. This variance includes both true inter‐patient variability and uncertainties inherent to the dose evaluation process: measurement, simulation, and unfolding. To isolate the true inter‐patient variability ($\sigma_{\mathrm{inter}}$), these uncertainty components were subtracted using the following equation:

(A3)
$$\sigma_{\mathrm{inter}}^{2}=\sigma_{\mathrm{total}}^{2}\;-\frac{1}{N}\sum_{i=1}^{N}\sigma_{\mathrm{intra},i}^{2}$$

where $\sigma_{\mathrm{total}}$ is the observed variance of the evaluated dose across patients, and the second term represents the mean squared intra‐patient uncertainty. The values of $\sigma_{\mathrm{intra}}$ were determined using a Monte Carlo approach with 10 000 samples per patient. Table A2 summarizes the uncertainty budget for the dose evaluation. The calculated $\sigma_{\mathrm{inter}}$ was negative (i.e., $\sigma_{\mathrm{intra}}>\sigma_{\mathrm{total}}$) for all locations except for the chest and abdomen. The main reason was the amplification of the uncertainties of measurement and simulation during the unfolding process, which resulted from the ill‐conditioned response matrices. Therefore, reliable isolation of the $\sigma_{\mathrm{inter}}$ was unavailable with our approach. Priorities for future work include improving counting statistics, particularly for the Al activation foil, and employing activation foils with more distinct neutron capture cross‐sections to reduce the condition number of the response matrix.

**TABLE A2:** Uncertainty budget of dose evaluation in this study.

Source	Type	Value	Remarks
Measurement			
Counting statistics	A	*C* ^−0.5^	Poisson statistics measured counts *C*.
Systematic uncertainty	B	Au, Mn, Al: 5%,^39^ TLD: 20%^40^	Including energy calibration, detector efficiency etc.
Simulation			
Nuclear data	B	Au: 2.0% Mn: 5.0% Al: 1.0%	Conservatively evaluated based on the JENDL nuclear data library.^41^
Calculation statistics	A	Evaluated by PHITS	Calculated by PHITS. Depending on the number of histories.
Intra‐patient ($\sigma_{\mathrm{intra}}$)		Evaluated by MC (*N* = 10 000)	Includes unfolding effects; location‐dependent
Inter‐patient ($\sigma_{\mathrm{inter}}$)		Not resolvable	*σ*_intra_ exceeded *σ*_total_ at most locations
